# Antibacterial Activity of *Fructus forsythia* Essential Oil and the Application of EO-Loaded Nanoparticles to Food-Borne Pathogens

**DOI:** 10.3390/foods5040073

**Published:** 2016-10-29

**Authors:** Na Guo, Qing-Yan Gai, Jiao Jiao, Wei Wang, Yuan-Gang Zu, Yu-Jie Fu

**Affiliations:** 1 Key Laboratory of Forest Plant Ecology, Ministry of Education, Northeast Forestry University, 150040 Harbin, China; guona_910817@163.com (N.G.); gqynefu@163.com (Q.-Y.G.); jjnefu@163.com (J.J.); wwnefu@126.com (W.W.); zyg_orl@126.com (Y.-G.Z.); 2 Engineering Research Center of Forest Bio-preparation, Ministry of Education, Northeast Forestry University, 150040 Harbin, China; 3 Collaborative Innovation Center for Development and Utilization of Forest Resources, Northeast Forestry University, 150040 Harbin, China

**Keywords:** *Forsythia suspense*, *Fructus forsythia* essential oil, chitosan nanoparticles, antibacterial activity, antibacterial mechanism

## Abstract

*Fructus forsythia* essential oil (FEO) with excellent antibacterial activity was rarely reported. The objective of the present study was to investigate the antibacterial activity and the antibacterial mechanism of FEO against two food-borne pathogenic bacteria, *Escherichia coli* (*E. coli*) and *Staphylococcus aureus* (*S. aureus*) in vitro. When treated FEO, the zones of inhibition (ZOI) of *E. coli* (20.5 ± 0.25 mm) and *S. aureus* (24.3 ± 0.21 mm) were much larger than control (*p* < 0.05). The minimum inhibitory concentrations (MICs) of FEO were 3.13 mg/mL and 1.56 mg/mL for *E. coli* and *S. aureus*, respectively. The antibacterial mechanism of FEO against *E. coil* was due to the changes in permeability and integrity of cell membrane leading to the leakage of nucleic acids and proteins. With the superior antibacterial activity of FEO, the nano-encapsulation method has been applied in FEO. When compared to FEO and blank chitosan nanoparticles, FEO-loaded nanoparticles (chitosan to FEO of 1:1) can effectively inhibit the growth of *E. coil* above 90% at room temperature. It is necessary to consider that FEO and FEO-loaded nanoparticles will become promising antibacterial additives for food preservative, cosmetic, and pharmaceutical applications.

## 1. Introduction

The presence and multiplication of microorganisms in a food system may cause an important problem, which can not only reduce the quality and quantity of food products [1], but also generate the illness and disease [2,3]. Since *Escherichia coli* (*E. coli*) was recognized as a food-borne pathogen in 1982, it had been found in variety of foodstuffs [4]. *Staphylococcus aureus* (*S. aureus*) was also considered a common pathogen which produced the staphylococcal heat-stable enterotoxins [5]. Food poisoning by *E. coli* and *S. aureus* is a growing increase problems in both developing and developed countries. Under these conditions, food preservatives come to prevent food decay and maintain the freshness of the food [6]. Chemical preservatives which are synthesized in a rapid way are generally applied to food preservatives. However, it has been proven that some chemical preservatives had undesirable biological effects in animals and humans leading to immeasurable risks [7]. Currently, compared to chemical preservatives, there is a growing interest to use safety and natural antibacterial compounds, particularly those from plants and fruits for the food preservation [8].

Essential oils (EOs), belonging to the secondary metabolism of scented plants, are the odorous and volatile products, which are obtained from flowers, leaves, seeds, bark, fruits, and roots [9]. They are generally recognized as safe (GRAS) substances with significant biological activity, such as anti-inflammatory, antioxidant, expectorant, carminative, pesticidal, and antimicrobial properties [10]. A great number of essential oils have been reported to possess the antibacterial activity, and some of them have been applied in food preservatives [11,12,13].

*Forsythia suspense* [(Thunb.) Vahl (Oleaceae)] is a valuable plant that is widely distributed throughout China, Korea, Japan, and European countries, which is used for both edible and medicinal properties [14]. *Fructus forsythia*, the mature fruit of *F. suspense* and *F. forsythiae* shell is also known as a famous traditional Chinese edible plan called “Lianqiao”. It can not only be used as a food flavoring additive, but also possess many biological activities [15,16,17]. *Fructus forsythia* essential oil (FEO) extracted from *Fructus forsythia* has been reported to have receivable antioxidant and antibacterial activity [18].

However, as a member of the essential oils, FEO is very easy to evaporate or decompose during food processing and drug formulation, especially under immediate heat, pressure, light, or oxygen conditions [19]. On this occasion, the emerging nano-encapsulation technique has been recently applied in food and pharmaceutical industries, which can improve the stability of products and lower the susceptibility of bioactive compounds during process and storage. In recent years, chitosan with the characteristics of biocompatibility, low toxicity, and biodegradability is getting more and more popular in nano-encapsulation [20]. On this basis, the ionic gelation technique based on the combining with the positively charged primary amino groups of chitosan and the negatively charged groups of polyanion—such as sodium tripolyphosphate (TPP)—can successfully encapsulate essential oils, proteins, genes, vitamins, and other hydrophilic or hydrophobic compounds [21].

The antibacterial activity of essential oil from the *Fructus forsythia* has been reported [18]. However, to the best of our knowledge, little is known on the antibacterial mechanism of FEO against some food-borne bacteria, and few studies focused on the applications of FEO-loaded nanoparticles in antibacterial system. The aim of the present study was to investigate the possible mechanism of FEO against *E. coli* and *S. aureus* and then produce FEO-loaded nanoparticles to improve the utilization, stability, and efficacy of the FEO. It can provide the scientific data for FEO as an alternative natural additive in food preservative, cosmetic, and pharmaceutical industries.

## 2. Materials and Methods

### 2.1. Materials

*Fructus forsythia* essential oil (FEO) (purity ≥ 95%) was isolated from the *Forsythia suspense* [(Thunb.) Vahl (Oleaceae)] by our group. Ciprofloxacin (purity > 98%, CAS # 85721-33-1), medium molecular weight chitosan (90%–95% degree of deacetylation, CAS # 9012-76-4), TPP (CAS # 7758-29-4), and Tween 80 (CAS # 9005-65-6) were purchased from Sigma–Aldrich (St. Louis, MO, USA). Crystal violet was obtained from Sigma Chemicals (Shanghai, China). Acetic acid (CAS # 64-19-7) was supplied by Aladdin Chemicals Co. (Shanghai, China). 30% Acr-Bis (29:1), Tris-HCl, pH 8.8, Tris-HCl, pH 6.8, 10% SDS, ammonium persulfate and TEMED were purchased from Beyotime Institute of Biotechnology(Beijing, China) and stored at 4 °C except 10% ammonium persulfate, which was stored at −20 °C.

### 2.2. Bacteria Cultures

Two food-borne bacteria, obtained from the Institute of Applied Microbiology, Heilongjiang Academy of Science (China), were used in this study: Gram-negative *E. coli* (ATCC 8739) and Gram-positive *S. aureus* (ATCC 6538). The bacterial strains were maintained on the agar plates at 4 °C and subcultured once a month. *E. coli* and *S. aureus* were activated in nutrient broth at 37 °C to a mid-log phase overnight. Before each experiment, the turbidity of the cell suspensions was measured at 600 nm and adjusted to the required concentration using the McFarland standard [22].

### 2.3. Antibacterial Activity

#### 2.3.1. Measurement of ZOI

The antibacterial activity of FEO against *E. coli* and *S. aureus* was tested by the inhibition zone assay [23]. Sterile discs (6 mm diameter) impregnated with essential oil (3 mg/disc) were air dried and placed on the seeded agar plates. 3 μg Ciprofloxacin per disc was taken as a positive control and sterile 0.9% NaCl solution in 0.5% Dimethyl sulfoxide (DMSO) was taken as blank control. The plates were incubated at 37 °C for 24 h. After incubation, the diameter of the transparent inhibition zone was measured. The experiments were conducted in triplicate under the same conditions.

#### 2.3.2. Measurement of MICs and MBCs

MIC and MBC values were determined using the serial two-fold dilutions method with some modifications [24]. FEO were dissolved in DMSO (0.5% (*v*/*v*)) and serially two folds diluted to the final concentration from 0.78 mg/mL to 50 mg/mL. The same volumes of activated *E. coli* and *S. aureus* suspension were co-cultured with the essential oil at 37 °C for 24 h. The MIC was defined as the lowest concentration of mixture which inhibited the growth of bacteria. The MBC was defined as the lowest concentration where no visible growth of bacteria was on the agar plate after incubation. Ciprofloxacin was used as positive control.

#### 2.3.3. Time-Kill Curves

Time-kill assays of *E. coli* and *S. aureus* treated by FEO (corresponding to control, MIC and 2 MIC) was determined the number of viable cells through a colony counting method in plates [25]. Time–kill curves were constructed by plotting log_10_CFU/mL against time (h).

### 2.4. Antibacterial Mechanism

#### 2.4.1. Scanning Electron Microscope Assay

The changes in bacterial morphology induced by FEO were observed by scanning electron microscopy (SEM) [26]. Overnight *E. coli* cultures were adjusted to 10^6^ CFU/mL using McFarland standard and treated with FEO at different concentrations (corresponding to control, MIC, and 2 MIC) for 4 h. After incubation, the suspensions were centrifuged at 1500g/min for 10 min and washed twice with 0.1 M phosphate buffer solution (PBS, pH 7.4). Then *E. coli* was fixed in 5% glutaraldehyde for 4 h in dark place. After three washes with PBS, all samples were dehydrated in a sequential graded ethanol (30%, 50%, 70%, 90%, and 100%). Finally, they were sputter-coated with platinum before SEM (Quanta 200 environmental scanning electron microscope system (FEI Company, Hillsboro, OR, USA)).

#### 2.4.2. Crystal Violet Assay

The alteration in membrane permeability was determined by crystal violet assay [27]. After incubation in LB medium at 37 °C for 24 h, *E. coli* and *S. aureus* were harvested and washed with PBS (pH 7.4) twice. The pellets were resuspended in sterile 0.9% NaCl solution and mixed with FEO at the concentration from 1/4MIC to 2MIC for 4 h. Cells then incubated with 10 μg/mL crystal violet in the dark for 15 min. After centrifuging, the absorbance of supernatant was determined by measuring the OD 590 nm using a UV-VIS spectrophotometer. The absorbance of crystal violet solution was considered as 100%. The crystal violet uptake was calculated using following formula:

% of takeup = (OD of the sample)/(OD of the crystal violet solution) × 100

#### 2.4.3. Measurement of Electrical Conductivity

The leakage of bacterial cells was investigated by measuring the leakage of electrolytes into the incubation medium with a conductivity meter (DDS-307, Precision & Scientific Instrument Co. Ltd., Shanghai, China) according to the method described by [28]. After incubation, the electric conductivities of *E. coli* and *S. aureus* suspension was adjusted to the same level of 5% glucose as isotonic bacteria. The FEO mixed with bacteria suspension with different concentrations (corresponding to control, MIC and 2 MIC) were measured the electric conductivities immediately, which was marked as L1. Then the conductivities of the mixtures were measured at 1, 2, 3, 4, 5, 6, 7, and 8 h marked as L2, respectively. The conductivities of boiled bacteria in 5% glucose were marked as L0. Untreated bacteria were served as the control. The relative electric conductivities were calculated using following formula:

Relative conductivity (%) = (L2 - L1) L0 × 100

#### 2.4.4. Measurement the Leakage of Cytoplasmic Materials

The cell membrane integrity of *E. coli* and *S. aureus* was examined by measuring the release of cell constituents into the supernatant [29]. The suspensions of bacteria were prepared as described above. Different concentrations of FEO (corresponding to control, MIC and 2MIC) were mixed. Untreated bacteria were served as the control. The loss of materials absorbing at 260 nm after 4 h was determined with a UV-VIS spectrophotometer. Correction was made for the absorption of the suspension with the same corresponding concentration of FEO after 2 min with two strains. The untreated cells were corrected with 0.9% NaCl solution in 0.5% DMSO, and the concentrations of proteins in supernatant were determined according to the method described by Xu [30].

#### 2.4.5. SDS-PAGE of Whole-Cell Protein

The degradations of *E. coli* and *S. aureus* proteins after FEO treatment were determined by SDS-PAGE [31]. The activated bacteria were treated with FEO at different concentrations (corresponding to control, MIC and 2MIC) at 37 °C for 4 h. After treatment, the suspension were washed twice with PBS buffer and a BCA protein assay kit was used to measure the concentrations of whole-cell protein lysates. Then, the suspension was resuspended in 100 μL of the loading buffer (0.06 M Tris-HCl (pH 6.8), 5% mercaptoethanol, 10% glycerol, 2% SDS, and 0.001% bromophenol blue). The mixtures were boiled for 10 min. The SDS-PAGE was performed with a 5% stacking gel and a 12% separating gel. The gel was then dyed with silver staining.

### 2.5. The Antibacterial Activity of FEO-Loaded Nanoparticles

#### 2.5.1. Preparation of FEO-Loaded Nanoparticles

FEO-loaded chitosan particles were prepared according to the method modified from [32]. CS/TPP nanoparticles were prepared according to the ionotropic gelation process. Briefly, chitosan solution (0.2%, 40 mL) was prepared by dissolving in aqueous acetic acid solution (1% *v*/*v*) at room temperature overnight. Tween 80 (1% *v*/*v*) was then added to the solution and stirred at 60 °C for 2 h to obtain a homogeneous mixture. FEO (0, 40 mg and 80 mg) was dissolved in CH_2_Cl_2_ (4 mL) and homogenized at a speed of 10,000 rpm for 3 min to obtain an oil-in-water emulsion. TPP solution was then dropped into the oil/water emulsion for 1 h. Thereafter, the prepared nanoparticles were isolated by centrifuging at 9000 rpm for about 30 min at 25 °C and washed severally with distilled water (20 mL). Finally, the obtained nanoparticles were stored at 4 °C. The as-prepared nanoparticles with weight ratios of chitosan to FEO (Chitosan: FEO) of 1:0, 1:0.5, and 1:1 were used for the present study.

#### 2.5.2. In Situ Antibacterial Assay of Nanoparticles

The antibacterial properties of FEO and nanoparticles were evaluated with the method according to Bukvički et al. with some modifications [33]. Prepared nanoparticles with chitosan to FEO of 1:0, 1:0.5, and 1:1 were thoroughly mixed with equal amounts of activated *E. coli* and *S. aureus* cells, respectively. Experimental teams were divided in three groups: one group was kept at 50 °C, the other at 25 °C, and the last one at the 4 °C. The inhibition percentage at 50 °C, 25 °C (1), and 4 °C (2) were calculated by optical density measured by ELISA plate reader using the following equations:

%inhibition = [(ODsample − OD0sample)/(ODgrowth* − OD0growth*) − (ODbland − OD0blank)] × 100(1)

%inhibition = [(ODsample − OD0sample)/(ODgrowth* − OD0growth*) − (ODbland − OD0blank)] × 100 − Tinhibition(2)

where OD0sample and ODsample corresponded to the absorbance of the strain growth in the presence of the nanoparticles before and after incubation at 612 nm, respectively; OD0blank and ODblank corresponded to the broth medium with dissolved compound before and after incubation, respectively; and OD0growth* and ODgrowth* to the strain growth in the absence of the nanoparticles before and after incubation at 50 °C and 25 °C, respectively. TInhibition corresponded to temperature inhibition of cells at 4 °C, measured according to the formula (3):

Tlnhibition = 100 − (ODTgrowth)/(ODT0growth) × 100(3)

where ODT0growth and ODTgrowth presented the growth of strain at 4 °C in medium, before and after incubation, respectively.

### 2.6. Statistical Analysis

All values were expressed as mean ± standard deviation (SD) of three experiments. Data were analyzed by using one-way ANOVA test. The photographs of ZOI, SEM, and SDS-PAGE showed in figures were only the representative. In all cases, a value of *p* < 0.05 was considered statistically significant.

## 3. Results and Discussion

### 3.1. Determination of Antibacterial Activity of FEO

The antibacterial activities of FEO against *E. coli* and *S. aureus* were detected by the measurement of the zone of inhibition (ZOI). The diameters of the ZOI against food-borne bacteria were shown in Table 1. The results showed that, the diameters of the ZOI for FEO treated with 3 mg/dis FEO were 20.5 ± 0.25 mm for *E. coli* and 24.3 ± 0.21 mm for *S. aureus*, respectively. Positive control (Ciprofloxacin) impregnated with 3 μg/dis were 20.1 ± 0.13 mm and 21.9 ± 0.17 mm, respectively. However, no ZOI was observed for negative control in the plate.

The minimum inhibitory concentration (MIC) and minimum bacterial concentration (MBC) of FEO were depicted in Table 1. The MIC value of FEO was 3.13 mg/mL against *E. coli* and 1.56 mg/mL against *S. aureus*, respectively. The MBC values were higher than the corresponding MIC values (Table 1). These results showed that the Gram-positive *S. aureus* appeared more sensitive than the Gram-negative *E. coli* toward FEO, which was in agreement with the findings of Oussalah who reported that the antimicrobial activities of plant essential oils were more preferred to gram-positive bacterium [34]. Possibly because that Gram-negative bacteria possessed an outer membrane surrounding the cell wall, which restricted the permeation of hydrophobic compounds through lipopolysaccharide coating [35].

### 3.2. Effect of FEO on the Rate of Kill of E. coil and S. aureus

**Figure 1:**
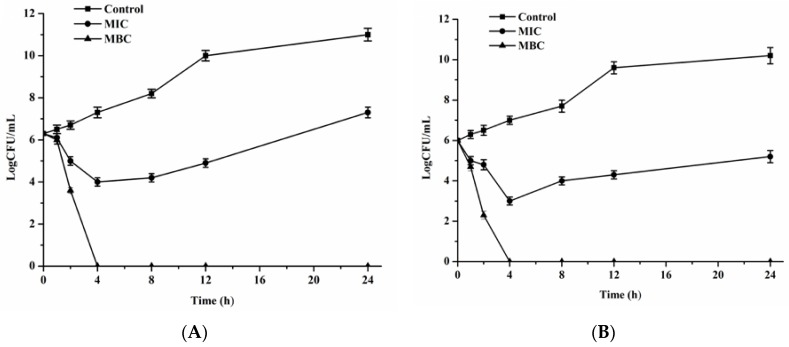
Time-kill kinetics of FEO against *E. coli* (**A**) and *S. aureus* (**B**). The concentrations used correspond to control, MIC, and MBC.

Further antibacterial activity of FEO was assessed by time-kill analysis, which was used to determine the speed of bactericidal activity of antibacterial agents [36]. As shown in Figure 1A,B, treatment with FEO exhibited a strong bactericidal effect on both *E. coli* and *S. aureus*, the bacterial population was inactivated within 4 h. A complete loss of viability was observed after 4 h exposure to FEO. These results suggested that the antibacterial activity of FEO was in a concentration- and time-dependent manner.

Recently, researchers had reported that the constituents of EOs such as terpenes terpenoids, phenylpropenes, carvacrol, and thymol could inhibit the multiplication of food-borne bacteria alone or synergistically [3,31]. It had been proven that there were total of 21 components in the FEO analyzed by GC–MS and monoterpene hydrocarbons and oxygenated monoterpenes were identified the major constituents in FEO [18,37]. Studies showed that oxygenated terpenoids such as alcoholic and phenolic terpenes had more remarkable antimicrobial activity than the other constituents. Many terpenes were known to a broad spectrum of antimicrobial activity, including bacteria and fungi [38]. It might be the main reason why FEO has a broad-spectrum and excellent antimicrobial activity.

### 3.3. Morphological Analysis of E. coli and S. aureus

**Figure 2:**
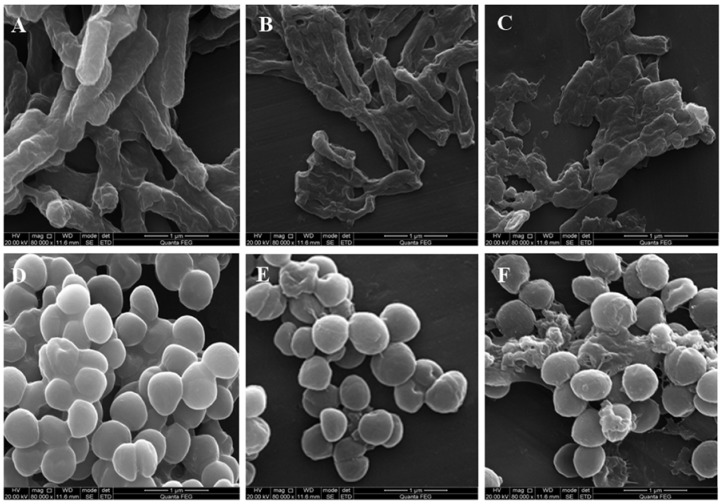
Scanning electron microphotographs of *E. coli* (**A**–**C**) and *S. aureus* (**D**–**F**). (**A**,**D**) images of untreated *E. coli* and *S. aureus*; (**B**,**E**) and (**C**,**F**) images of *E. coli* and *S. aureus* treated with different concentrations of FEO, correspond to MIC and 2MIC.

SEM was used to visually observe the morphology changes of *E. coli* and *S. aureus* cells after FEO treatment (Figure 2). The untreated *E. coli* cells showed the distinctive striated cell wall (Figure 2A). Their normal morphology (rod shape) was retained. After 4 h of MIC treatment, the surface of the *E. coli* cell was wrinkled and irregular (Figure 2B). At the 2MIC level, some cells were damaged, lysing to debris (Figure 2C). As for *S. aureus* cells, the untreated bacteria presented the smooth cell membrane (Figure 2D). In contrast, *S. aureus* cells treated with FEO at MIC level showed the destruction of the morphology (Figure 2E). Furthermore, it was obvious to observe that the surface of the cell membrane was collapsed, broken, and lysed at 2MIC level treatment (Figure 2F).

The morphological alterations in the structure of cell wall and cell membrane were investigated by SEM analysis. The severe morphological alterations on the cells confirmed the FEO possessed excellent antibacterial activities. Meanwhile, the changes of the cell morphology demonstrated the effect of FEO on membrane permeability and integrity. It led to the damage of the bacterial cell wall and cell membrane, and the leakage of intracellular materials was outflowed to the surrounding environment. The results of SEM graphs were in agreement with some research that reported that essential oil could lyse cell walls and cell membranes with decreases of heterogeneity electron density in cytoplasm [31].

### 3.4. Crystal Violet Study

**Figure 3:**
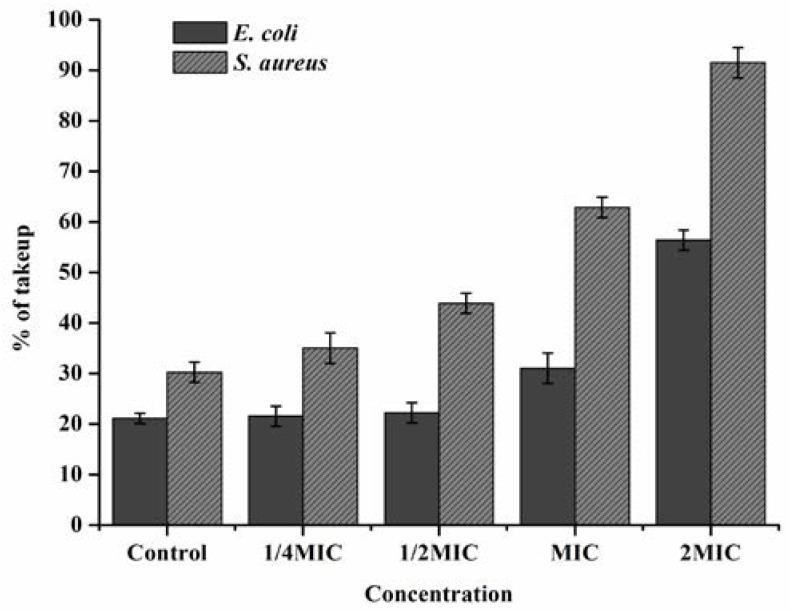
Effect on membrane permeability of *E. coil* and *S. aureus* treated with FEO. The concentrations were corresponded to control, 1/4MIC, 1/2MIC, MIC, and 2MIC (MBC). Values of each curve are means ± SD (*n* = 3).

A number of authors had mentioned the antimicrobial activity and antimicrobial mechanism of EOs. It was generally believed that EOs changed the cell cytoplasmic membrane of microorganism. Crystal violet which was generally poorly penetrated the membrane, could easily enter the damaged cell membrane [39]. A significant enhancement in the uptake of crystal violet was observed in Figure 3, the uptake of crystal violet of *E. coli* and *S. aureus* were 21.1% and 30.2% in the absence of FEO respectively, increased their signal to 56.4% and 91.5% after FEO treatment at 2MIC.

### 3.5. Relative Electrical Conductivity Study

**Figure 4:**
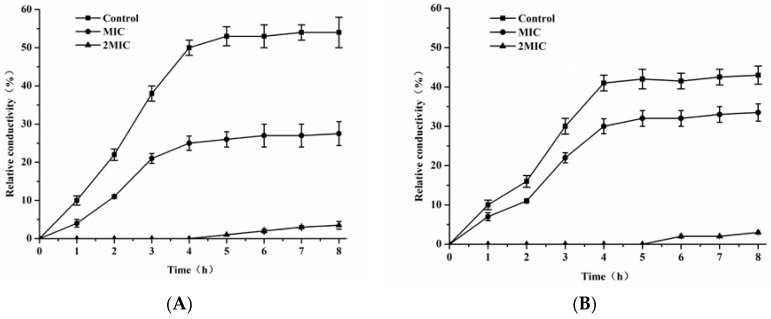
Effect of FEO on cellular leakage of *E. coil* (**A**) and *S. aureus* (**B**). The concentrations used corresponded to control, MIC, and 2MIC. Values of each curve are means ± SD (*n* = 3).

Further antibacterial mechanism of FEO against *E. coli* and *S. aureus* was confirmed with the measurement of the relative electric conductivity. As shown in Figure 4, the percentage of the relative electric conductivities of *E. coli* and *S. aureus* samples were increased to about 26.2% and 29.7% at the concentration of MIC in the first 4 h, and then reached a relative stable phage in the next 4 h. Compared to the MIC, the percentage of the relative electric conductivity of the suspensions were sharply reached to 50.2% and 41.3% in the first 4 h, and stabilized at a high level in the next 4 h at the concentration of 2MIC. The increase of the relative electric conductivity of samples treated with different concentrations of FEO suggested that the cytoplasmic membranes were disrupted, which caused the leakage of intracellular ingredient, especially losses of electrolytes including K^+^, Ca^2+^, Na^+^, and so on [40].

### 3.6. Leakage of Cytoplasmic Materials

The leakage of cytoplasmic material was considered as severe and irreversible damage to the cytoplasmic membrane. Measurement of specific cell leakage markers such as 260 nm absorbing materials and proteins could indicate the changes of membrane integrity compared to unexposed cells [41]. In Table 2, compared to control, after treatment with FEO at the concentration of MIC and 2MIC, the concentration of cell constituents (OD 260 nm) and proteins in suspensions increased 2.3, 10.2 times and 6.0, 16.0 times to *E. coil*, and 4.3, 12.9 times and 10.3, 19.7 times to *S. aureus*, respectively. These changes of the concentrations of nucleic acids and proteins suggested that essential oil damaged cytoplasmic membrane and subsequently caused leakage of intracellular constituents.

### 3.7. SDS-PAGE of Protein Patterns of Bacteria Treated with FEO

**Figure 5:**
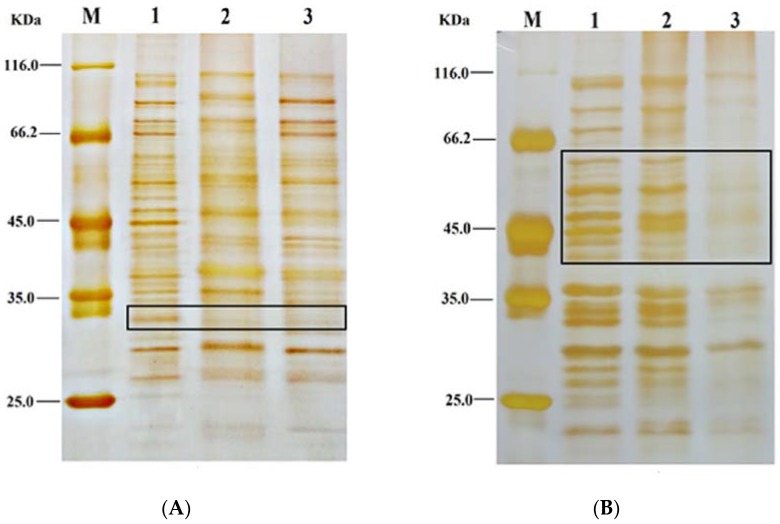
SDS-PAGE of whole proteins. (**A**): *E. coil*, Lane-1: protein marker; Lane-2: untreated control; Lane-3: MIC treatment and Lane-4: 2MIC treatment; (**B**): *S. aureus*, Lane-1: protein marker; Lane-2: untreated control; Lane-3: MIC treatment and Lane-4: 2 MIC treatment.

To further investigate the probable antibacterial mechanisms of FEO, the whole protein patterns of bacteria were analyzed. Compared with the National Center for Biotechnology Information, SDS-PAGE of whole-cell protein lysates was to identify the possible target proteins after FEO treatment. The results demonstrated that the membrane integrity was destructed after FEO treatment (Figure 5A,B). Lane 1, 2, 3, and 4 were corresponding to maker, control, MIC, and 2MIC when treated with FEO against *E. coli* (A) and *S. aureus* (B), respectively. The protein profiles of bacteria treated with FEO differed from those of the control. Compared with the untreated cells, the bands approximately at 60 kDa, 52 kDa, 33 kDa, and 28 kDa were degraded in *E. coli* samples. Whereas bands at about 30 kDa and 26 kDa were also degraded in *S. aureus* samples. Outer membrane protein A (OmpA: 28 kDa) was critical for the integrity of the bacterial membrane as a physical linkage with peptidoglycan layer in *E. coli* [42]. The membrane-associated protein TcaA (TcaA: 52 kDa) also played a critical role in the synthesis of cell wall in *S. aureus* [43]. The degradation and destruction of the critical functional proteins led to inactivation of the microorganism. More proteins associating with the damage of the membrane needed to be advanced investigations.

### 3.8. The Application of FEO-Loaded Nanoparticles Against E. coli and S. aureus

The encapsulation of functional EOs in emulsions had been applied as a popular method to improve the utilization and stability of EOs [20]. Nano-emulsions which had the advantage of small particle size, low turbidity, and controlled ease of functional attributes had been appropriate for commercial applications, including preservatives in foods, beverages, cosmetics, and pharmaceuticals [44,45,46,47]. These EO-loaded nanoparticles were usually prepared by ionic gelation technique. In my study, the FEO-loaded nanoparticles were prepared with the positively charged primary amino groups of chitosan and the negatively charged groups TPP. This nano-encapsulation had a good effect on inhibiting the growth of two food-borne pathogens, *E. coli* and *S. aureus*. The result was presented in Table 3, the antimicrobial activity of FEO-loaded nanoparticles against *E. coli* and *S. aureus* was better in the refrigerated conditions. With the highest tested concentrations (chitosan to FEO of 1:1) 100% inhibition was achieved in 36 h at 4 °C. Inhibition percentage was quiet constant for all the concentrations used, during the storage period at a temperature of 4 °C, which was in the range from 98.43% to 95.29%. At room temperature, FEO-loaded nanoparticles (chitosan to FEO of 1:0.5 and 1:1) could also effectively inhibit the growth of the food-borne pathogens above 50%. With the temperature reached to 50 °C, the inhibition rate of pathogens with FEO-loaded nanoparticles were reduced, which was caused by the significant effect of the physical properties of oil and water system under high temperature. The increased temperature resulted in the increased release rate and diffusion coefficient of core material [48]. Compared to blank nanoparticles, encapsulation of essential oils in nano-systems represented an efficient approach to increase the physical stability of bioactive compounds and to protect them from undesirable changes.

## 4. Conclusions

The results of the present study indicate that FEO is a potential antibacterial compound against two food-borne bacteria. The conversion of *Fructus forsythia* essential oil into nano-emulsions can greatly enhance the antibacterial activity against food-borne pathogenic bacteria. The antibacterial mechanism of FEO is the disruption of the cell membrane which changes the cell membrane permeability, and leads to the release of some cellular components such as ions, DNA, and proteins. Also, the antibacterial mechanism of FEO may degrade bacterial proteins. The present work provides strong evidence that the essential oil of *Fructus forsythia* is available as a green effective antibacterial additive. Further studies are needed to evaluate the efficacy and safety of EO-loaded nanoparticles in suitable food systems.

## Figures and Tables

**Table 1:** Antibaterial activity (ZOI, MIC, and MBC) of the FEO (mean ± SD).

Bacteria	FEO			Ciprofloxacin		
	*D/mm* ^a^	MIC ^b^	MBC ^c^	*D/mm*	MIC	MBC
*E. coli*	20.5 ± 0.25	3.13	6.25	20.1 ± 0.13	3.13	3.13
*S. aureus*	24.3 ± 0.21	1.56	3.13	21.9 ± 0.17	1.56	1.56

^a^ ZIO (mm) impregnated with 3 mg of *Fructus forsythia* essential oil or 3 μg of Ciprofloxacin per discs (6 mm); ^b^ MIC, minimal inhibitory concentrations (FEO, mg/mL; Ciprofloxacin, μg/mL); ^c^ MBC, minimal bactericidal concentration (FEO, mg/mL; Ciprofloxacin, μg/mL).

**Table 2:** Effects of the FEO on cellular of leakage *E. coil* and *S. aureus*.

Bacteria	Cell Constituents’ Release					
	Protein (ug/mL)			Cell Constituents(OD 260 nm)		
	Control	MIC	2MIC	Control	MIC	2MIC
*E. coil*	9.3 ± 1.3	21.7 ± 4.7	64.7 ± 7.5	0.022 ± 0.007	0.224 ± 0.031	0.351 ± 0.040
*S. aureus*	10.5 ± 1.7	40.8 ± 5.0	108.1 ± 9.4	0.031 ± 0.012	0.417 ± 0.041	0.612 ± 0.031

Values represent means of three independent replicates ± SD.

**Table 3:** Effects of FEO-loaded nanoparticles against *E. coli* and *S. aureus*.

Bacteria	Chitosan: FEO (*w*/*w*)	Temp. (°C)	Percentage of Inhibition of Stains with Nanoparticles				
			0 h	12 h	24 h	36 h	48 h
*E. coil*	1:0	+50	0.00 ± 0.00	19.13 ± 3.23	13.14 ± 4.12	10.21 ± 3.89	6.21 ± 4.21
		+25	0.00 ± 0.00	71.23 ± 2.31	60.67 ± 3.24	38.13 ± 2.16	15.80 ± 2.40
		+4	0.00 ± 0.00	97.17 ± 1.29	96.89 ± 0.81	96.19 ± 1.33	95.29 ± 1.71
	1:0.5	+50	0.00 ± 0.00	34.14 ± 3.29	27.22 ± 1.79	20.11 ± 4.28	10.12 ± 3.15
		+25	0.00 ± 0.00	84.02 ± 2.14	73.13 ± 2.17	60.59 ± 3.12	51.21 ± 2.18
		+4	0.00 ± 0.00	98.43 ± 0.53	98.01 ± 0.19	96.12 ± 0.67	95.31 ± 1.04
	1:1	+50	0.00 ± 0.00	73.19 ± 2.89	50.81 ± 4.12	39.02 ± 2.10	12.03 ± 3.21
		+25	0.00 ± 0.00	100.00 ± 0.00	97.02 ± 1.02	93.01 ± 2.11	90.12 ± 2.10
		+4	0.00 ± 0.00	100.00 ± 0.00	100.00 ± 0.00	100.00 ± 0.00	98.46 ± 0.51
*S. aureus*	1:0	+50	0.00 ± 0.00	21.89 ± 3.09	17.34 ± 2.78	9.22 ± 2.90	6.78 ± 5.12
		+25	0.00 ± 0.00	84.55 ± 2.40	72.38 ± 3.07	54.09 ± 3.02	33.93 ± 4.02
		+4	0.00 ± 0.00	98.09 ± 1.03	96.47 ± 1.09	95.33 ± 0.48	95.01 ± 1.27
	1:0.5	+50	0.00 ± 0.00	41.23 ± 5.63	37.87 ± 2.99	31.48 ± 5.09	21.41 ± 2.97
		+25	0.00 ± 0.00	93.77 ± 2.66	87.40 ± 2.22	80.05 ± 7.11	70.32 ± 3.78
		+4	0.00 ± 0.00	100.00 ± 0.00	98.12 ± 1.32	96.14 ± 2.02	95.11 ± 1.04
	1:1	+50	0.00 ± 0.00	81.34 ± 3.48	70.01 ± 4.22	53.26 ± 2.99	31.06 ± 4.30
		+25	0.00 ± 0.00	100.00 ± 0.00	100.00 ± 0.00	100.00 ± 0.00	98.21 ± 2.77
		+4	0.00 ± 0.00	100.00 ± 0.00	100.00 ± 0.00	100.00 ± 0.00	100.00 ± 0.00

Values represent means of three independent replicates ± SD.

## References

[1] Soliman K.M., Badeaa R.I. (2002). Effect of oil extracted from some medicinal plants on different mycotoxigenic fungi. Food Chem. Toxicol..

[2] Jacob C., Mathiasen L., Powell D. (2010). Designing effective messages for microbial food safety hazards. Food Control.

[3] Patra J.K., Baek K.H. (2016). Antibacterial activity and action mechanism of the essential oil from *Enteromorpha linza* L. against foodborne pathogenic bacteria. Molecules.

[4] Kornacki J.L., Marth E.H. (1982). Foodborne illness caused by *Escherichia coli*: A review. J. Food Prot..

[5] Souza E.L.D., Barros J.C.D., Conceição M.L.D., Gomes Neto N.J., Costa A.C.V.D. (2009). Combined application of *Origanum vulgare* L. essential oil and acetic acid for controlling the growth of *Staphylococcus aureus* in foods. Braz. J. Microbiol..

[6] Klein G., Ruben C., Upmann M. (2013). Antimicrobial activity of essential oil components against potential food spoilage microorganisms. Curr. Microbiol..

[7] Osman K.A., Abdulrahman H.T. (2003). Risk assessment of pesticide to human and the environment. Saudi J. Biol. Sci..

[8] Tiwari B.K., Valdramidis V.P., O’Donnell C.P., Muthukumarappan K., Bourke P., Cullen P.J. (2009). Application of natural antimicrobials for food preservation. J. Agric. Food Chem..

[9] Burt S. (2004). Essential oils: Their antibacterial properties and potential applications in foods—A review. Int. J. Food Microbiol..

[10] Hyldgaard M., Mygind T., Meyer R.L. (2012). Essential oils in food preservation: Mode of action, synergies, and interactions with food matrix components. Front. Microbiol..

[11] Pirbalouti A.G., Hashemi M., Ghahfarokhi F.T. (2013). Essential oil and chemical compositions of wild and cultivated *Thymus daenensis Celak* and *Thymus vulgaris* L.. Ind. Crops Prod..

[12] Moazeni N., Khajeali J., Izadi H., Mahdian K. (2014). Chemical composition and bioactivity of *Thymus daenensis Celak* (Lamiaceae) essential oil against two lepidopteran stored-product insects. J. Essent. Oil Res..

[13] Zengin H., Baysal A.H. (2014). Antibacterial and antioxidant activity of essential oil terpenes against pathogenic and spoilage-forming bacteria and cell structure-activity relationships evaluated by SEM microscopy. Molecules.

[14] Sun Q.Q., Jiang Z.T., Li R. (2012). The research progress of natural preservatives forsythia oil. China Food Addit..

[15] Kang H.S., Lee J.Y., Kim C.J. (2008). Anti-inflammatory activity of arctigenin from *Forsythiae Fructus*. J. Ethnopharmacol..

[16] Ozaki Y., Rui J., Tang Y.T. (2000). Antiinflammatory effect of *Forsythia suspensa* VAHL and its active principle. Biol. Pharm. Bull..

[17] Qu H., Zhang Y., Wang Y. (2008). Antioxidant and antibacterial activity of two compounds (*forsythiaside* and *forsythin*) isolated from *Forsythia suspense*. J. Pharm. Pharmacol..

[18] Jiao J., Fu Y.J., Zu Y.G., Luo M., Wang W., Zhang L., Li J. (2012). Enzyme-assisted microwave hydro-distillation essential oil from *Fructus forsythia*, chemical constituents, and its antimicrobial and antioxidant activities. Food Chem..

[19] Chalier P., Arfa A.B., Preziosi-Belloy L., Gontard N. (2007). Carvacrol losses from soy protein coated papers as a function of drying conditions. J. Appl. Polym. Sci..

[20] Donsì F., Annunziata M., Sessa M., Ferrari G. (2011). Nanoencapsulation of essential oils to enhance their antimicrobial activity in foods. LWT Food Sci. Technol..

[21] Shu X.Z., Zhu K.J. (2000). A novel approach to prepare tripolyphosphate/chitosan complex beads for controlled release drug delivery. Int. J. Pharm..

[22] Firuzi O., Asadollahi M., Gholami M., Javidnia K. (2010). Composition and biological activities of essential oils from four Heracleum species. Food Chem..

[23] Kim J., Marshall M.R., Wei C.I. (1995). Antibacterial activity of some essential oil components against five foodborne pathogens. J. Agric. Food Chem..

[24] Ogata M., Hoshi M., Urano S., Endo T. (2000). Antioxidant activity of eugenol and related monomeric and dimeric compounds. Chem. Pharm. Bull..

[25] Zu Y.G., Liu X.L., Fu Y.J., Wu N., Kong Y., Wink M. (2000). Chemical composition of the SFE-CO_2_ extracts from *Cajanus cajan* (L.) Huth and their antimicrobial activity in vitro and in vivo. Phytomedicine.

[26] Yong A.L., Ooh K.F., Ong H.C., Chai T.T., Wong F.C. (2015). Investigation of antibacterial mechanism and identification of bacterial protein targets mediated by antibacterial medicinal plant extracts. Food Chem..

[27] Vaara M., Vaara T. (1981). Outer membrane permeability barrier disruption by polymyxin in polymyxin-susceptible and-resistant *Salmonella typhimurium*. Antimicrob. Agents Chemother..

[28] Kong M., Chen X.G., Liu C.S., Liu C.G., Meng X.H., Yu L.J. (2008). Antibacterial mechanism of chitosan microspheres in a solid dispersing system against *E. coli*. Colloids Surf. B.

[29] Diao W.R., Hu Q.P., Zhang H., Xu J.G. (2014). Chemical composition, antibacterial activity and mechanism of action of essential oil from seeds of fennel (*Foeniculum vulgare* Mill.). Food Control.

[30] Xu J.G., Hu Q.P., Wang X.D., Luo J.Y., Liu Y., Tian C.R. (2010). Changes in the main nutrients, phytochemicals, and antioxidant activity in yellow corn grain during maturation. J. Agric. Food Chem..

[31] Mohammadi A., Hashemi M., Hosseini S.M. (2015). Nanoencapsulation of Zataria multiflora essential oil preparation and characterization with enhanced antifungal activity for controlling Botrytis cinerea, the causal agent of gray mould disease. Innov. Food Sci. Emerg. Technol..

[32] Bukvički D., Stojković D., Soković M., Vannini L., Montanari C., Pejin B., Marin P.D. (2014). Satureja horvatii essential oil: In vitro antimicrobial and antiradical properties and in situ control of *Listeria monocytogenes* in pork meat. Meat Sci..

[33] Oussalah M., Caillet S., Saucier L., Lacroix M. (2006). Antimicrobial effects of selected plant essential oils on the growth of a *Pseudomonas putida* strain isolated from meat. Meat Sci..

[34] Vaara M. (1992). Agents that increase the permeability of the outer membrane. Microbiol. Rev..

[35] Aiyegoro O.A., Afolayan A.J., Okoh A.I. (2009). In vitro antibacterial time kill studies of leaves extracts of Helichrysum longifolium. J. Med. Plants Res..

[36] Kordali S., Kotan R., Mavi A., Cakir A., Ala A., Yildirim A. (2013). Determination of the chemical composition and antioxidant activity of the essential oil of *Artemisia dracunculus* and of the antifungal and antibacterial activities of Turkish *Artemisia absinthium*, A. drancunculus, *Artemisia santonicum*, and *Artemisia spicigeraessential* oils. J. Agric. Food Chem..

[37] Zeng W.C., Zhu R.X., Jia L.R., Gao H., Zheng Y., Sun Q. (2011). Chemical composition, antimicrobial and antioxidant activities of essential oil from *Gnaphlium affine*. Food Chem. Toxicol..

[38] Griffin S.G., Wyllie S.G., Markham J.L. (1999). The role of structure and molecular properties of terpenoids in determining their antimicrobial activity. Flavour Fragr. J..

[39] Li N., Luo M., Fu Y.J., Zu Y.G., Wang W., Zhang L., Sun Y. (2013). Effect of Corilagin on Membrane Permeability of *Escherichia coli*, *Staphylococcus aureus* and *Candida albicans*. Phytother. Res..

[40] Dayan F.E., Watson S.B., Galindo J.C.G., Hernández A., Dou J., McChesney J.D., Duke S.O. (1999). Phytotoxicity of quassinoids: Physiological responses and structural requirements. Pestic. Biochem. Physiol..

[41] Bajpai V.K., Sharma A., Baek K.H. (2013). Antibacterial mode of action of *Cudrania tricuspidata* fruit essential oil, affecting membrane permeability and surface characteristics of food-borne pathogens. Food Control.

[42] Ried G., Henning U. (1987). A unique amino acid substitution in the outer membrane protein OmpA causes conjugation deficiency in *Escherichia coli* K-12. FEBS Lett..

[43] Brandenberger M., Tschierske M., Giachino P., Wada A., Berger-Bächi B. (2000). Inactivation of a novel three-cistronic operon *tcaR-tcaA-tcaB* increases teicoplanin resistance in *Staphylococcus aureu*. Biochem. Biophys. Acta.

[44] McClements D.J., Rao J. (2011). Food-grade nanoemulsions: Formulation, fabrication, properties, performance, biological fate, and potential toxicity. Crit. Rev. Food Sci..

[45] Solans C., Izquierdo P., Nolla J., Azemar N., Garcia-Celma M.J. (2005). Nano-emulsions. Curr. Opin. Colloid Interface Sci..

[46] Rao J., McClements D.J. (2012). Food-grade microemulsions and nanoemulsions: Role of oil phase composition on formation and stability. Food Hydrocoll..

[47] Quintão F.J., Tavares R.S., Vieira-Filho S.A., Souza G.H., Santos O.D. (2013). Hydroalcoholic extracts of Vellozia squamata: Study of its nanoemulsions for pharmaceutical or cosmetic applications. Rev. Bras. Farmacogn..

[48] Li P.H., Lu W.C. (2015). Effects of storage conditions on the physical stability of d-limonene nanoemulsion. Food Hydrocoll..

